# Rheumatoid peripheral blood phagocytes are primed for activation but have impaired Fc-mediated generation of reactive oxygen species

**DOI:** 10.1186/ar2144

**Published:** 2007-03-13

**Authors:** Anna-Marie Fairhurst, Paul K Wallace, Ali SM Jawad, Nicolas J Goulding

**Affiliations:** 1 William Harvey Research Institute, Barts and the London, Queen Mary's School of Medicine and Dentistry, Charterhouse Square, London EC1M 6BQ, UK; 2 Flow Cytometry Center, Roswell Park Cancer Institute, Elm and Carlton Streets, Buffalo, NY 14263, USA; 3 Department of Rheumatology, Barts and the London NHS Trust, Bancroft Road, Mile End, London E1 4DG, UK

## Abstract

Significant levels of circulating immune complexes (ICs) containing rheumatoid factors and immunoglobulin G in peripheral blood are a characteristic feature of rheumatoid arthritis (RA). ICs interact through Fcγ receptors (FcγR) to activate phagocytes in numerous inflammatory processes. The high concentration of neutrophils in synovial fluid during active phases of the disease, together with their destructive capacity, pose important questions as to their role in the pathogenesis of RA. Functional defects in RA or control peripheral blood neutrophil FcγRs were examined with a specific FcγR-mediated reactive oxygen species (ROS) assay. Heterologous cross-linking of FcγRIIa and FcγRIIIb on neutrophils resulted in a significantly decreased production of ROS by RA cells compared with controls matched for age and sex. However, expression and homologous ligation of receptors did not differ between these groups. These data suggest that neutrophil priming does occur before emigration into the joint and that blood neutrophils from patients with RA have a functional impairment in cooperative FcγR-mediated ROS generation. This may account for the increased susceptibility to bacterial infection that arises in patients with severe disease.

## Introduction

Immune complex (IC) formation is a characteristic feature of rheumatoid arthritis (RA). ICs have been located in the synovial fluid, the superficial layers of the cartilage and circulating in the periphery [1-3]. ICs activate inflammatory processes by two main overlapping mechanisms: first, through the soluble proteins of the complement system, and second, through interaction with one of three described receptors for the Fc constant region of immunoglobulin G (IgG), the Fcγ receptors (FcγR) [3-5]. IC interaction through FcγRs activates phagocytic neutrophils and mononuclear phagocytes in several inflammatory processes.

Both murine and human studies have provided evidence for a primary role of neutrophils in RA. Of the cells infiltrating the synovial fluid during the active phases of RA, 80 to 90% are neutrophils and turnover can exceed 10^9 ^cells per day in a 30 ml joint effusion [6,7]. Depletion of neutrophils in an experimental model of the disease prevents the development of inflammation and decreases it once it has ensued [8]. Activation of neutrophils leads to degranulation, phagocytosis and the generation of reactive oxygen species (ROS) [9,10]. The subsequent release of proteolytic enzymes and reactive oxygen metabolites can result in tissue damage [11,12].

Neutrophils express FcγRIIa (CD32a), which is a single-transmembrane receptor with its own immunoreceptor tyrosine-based activation motif (ITAM) in the intracellular domain, and FcγRIIIb (CD16b), which does not have a cytoplasmic tail but is inserted into the membrane by means of a glycosylphosphatidylinositol anchor [13,14]. This FcγRIII isotype is expressed exclusively on granulocytes. It is the most abundant FcγR present on neutrophils and it believed to be the primary binding molecule for ICs, working in tandem with FcγRIIa or complement receptor type 3(CR3; also referred to as CD11b/CD18 or Mac-1) to mediate a full inflammatory response. Despite the lack of an intracellular signalling domain, homotypic ligation may transduce signalling events that are distinct from homotypic FcγRIIa and heterologous ligation [15].

In addition, there is a large amount of evidence that FcγRIIIb is important in both IC-mediated activation and phagocytosis of opsonised bacteria. Several investigations have shown that inhibition or removal of this receptor restricts both insoluble and soluble IC-mediated activation [16-20]. However, the extent of FcγRIIIb involvement is subject to debate.

Allelic specificity of FcγRIIIb affects the efficiency of phagocytosis of opsonised bacteria [21,22]. FcγRIIIb exists as one of three serological allotypes: neutrophil antigen (NA)1, NA2 or SH-FcγRIIIb (also referred to as HNA-1a, HNA-1b and HNA-1c, respectively [23], in which NA1 and NA2 differ in five nucleotides and SH-FcγRIIIb differs from NA2 at a single base. FcRγIIIb-NA1 has been shown to mediate a higher response in the internalisation of erythrocytes, as well as in the phagocytosis of opsonised bacteria. There have been no significant associations between polymorphisms in FcγRIIIb and the development of disease; however, patients with RA who have the NA2 allele are associated with an increased prevalence of respiratory tract infections [24-27]. This suggests a mechanistic role for FcγRIIIb in the well-known increased susceptibility and increased risk of death from bacterial infection observed in RA [28-30].

The importance of the adhesion molecules, integrins and selectins in mediating the rolling and tethering of neutrophils to the endothelium is well established [31]. In this study we measured the expression of L-selectin (CD62L) and β-integrin, CR3, which are established markers of neutrophil activation [32,33]. The most accepted inflammatory measurements used in clinical medicine are the erythrocyte sedimentation rate (ESR) and levels of C-polysaccharide reactive protein (C-reactive protein; CRP) [34]. ESR indirectly reflects potentially increasing serum proteins, such as fibrinogen, acute-phase proteins and immunoglobulins [35]. CRP is the most studied acute-phase protein and is a good measure of activity of disease because high circulating levels are correlated with the acute inflammatory stages of the disease, and low levels with quiescent stages [36].

The destructive capacity of joint neutrophils in RA, together with a delay in apoptosis, is well established, but peripheral changes in neutrophil function are less clear. In this study we examined the expression and function of the individual FcγRs on neutrophils in patients with RA who had active synovitis. Although the basal and stimulated expression of FcγRIIa was similar to that of FcγRIIIb, heterologous ligation of both receptors resulted in a decrease in FcγR-mediated ROS generation in patients with RA. Although several studies have demonstrated that individual homologous or heterologous ligation of FcγRIIa and FcγRIIIb may induce ROS generation, this is the first report to demonstrate a deficiency in the co-ligation of these receptors in RA [17,37,38].

## Materials and methods

### Patients

Patients attending the Rheumatology Clinic at The Royal London Hospital, Mile End, London, UK, were diagnosed with RA ac the criteria outlined by the American College of Rheumatology (ACR (ARA) [39]). Of 18 patients with RA recruited for the investigations, 4 were male and 14 were female. Demographics of the patients are shown in Table 1. All were assessed as having active synovitis. Peripheral blood was collected into a syringe Vacutainer containing 3.8% EDTA (10% v/v; Becton Dickinson, Oxford, UK). The average age of all 36 volunteers was 62.6 ± 13.2 (mean ± SD). Venous blood samples from control volunteers matched for age and sex were taken within 1 hour of collection from the patient. All blood was taken with full consent and with prior approval from the local research ethics committee (East London and City Health Authority Ethics Committee). Circulating blood levels of haemoglobin, CRP, leucocytes and platelet counts, in addition to the ESR, were determined in the population with RA at the hospital where they were receiving treatment.

**Table 1:** RA patient demographic data, blood inflammatory parameters and treatment profile

Sex	Age	ESR (mm/h)	CRP (mg/l)	Hb (g/dl)	Platelets (× 10^9^/l)	WBC (× 10^9^/l)	Lymphocytes Neutrophils (× 10^9^/l)	(× 10^9^/)	Medication
M	71	58	60	12.7	351	10.3	7.8	1.7	Diclofenac
M	53	93	36	10.3	417	10.5	8.4	1.5	Methotrexate
M	78	57	74	12.4	422	7.8	5.5	1.5	-
M	62	16	17	14.1	207	6.1	3.5	2	-
F	61	37	-	12.3	261	6.1	3.9	1.6	Leflunomide
F	75	61	77	11.6	246	6.5	5.3	0.8	-
F	42	27	17	13.7	356	12.7	10.4	1.8	-
F	63	49	45	11.1	359	5.8	4.7	0.6	Clarithromycin
F	56	20	-	13.9	263	8	5	2.5	Methotrexate, prednisolone, Losec, perindopril, Sinemet, aspirin
F	82	37	-	12	195	8.2	5.6	1.8	-
F	63	41	41	11.4	309	9.8	9.6	3.3	-
F	57	-	13.9	-	-	-	-	-	Methotrexate, prednisolone, (depo-medrone)
F	76	57	72	11	244	7.7	6.3	0.9	-
F	49	-	-	11.4	204	7.2	4.4	2.5	Azothioprine, meloxicam
F	45	48	-	12.9	259	7.4	5.6	1.3	Methotrexate, indomethacin
F	92	1	-	13.9	232	9.6	7.5	0.9	Penicillamine
F	56	18	-	13.7	481	8.2	5.1	1.9	Methotrexate, folic acid, prednisolone, Vioxx, antihypertensive treatment
F	44	7	-	13.2	241	7.1	4.8	1.3	Leflunomide
F	68	12	5	12.7	284	6.9	4.6	1.7	Azothioprine, prednisolone, alendronate, thyroxine, Losec
F	56	18	23	13.7	481	8.2	5.1	1.9	-
F	78	12	5	-	-	-	-	-	-
F	52	16	11	-	-	-	-	-	Leflunomide

Eighteen patients (4 male, 14 female; age 62.6 ± 13.4 years (mean ± SD) with active synovitis and receiving a range of disease-modifying anti-rheumatic agents were recruited into the study. CRP, C-reactive protein; ESR, erythrocyte sedimentation rate; Hb, haemoglobin; WBC, white blood cells.

### Isolation of leucocytes by dextran sedimentation

Preliminary studies demonstrated that the surface expressions of L-selectin and CR3 were altered on cell separation by using density centrifugation methods (Percoll and Ficoll; data not shown). Dextran sedimentation produced minimal phenotypic cellular changes and controlled for serum immunoglobulin and differences in cell numbers. The expression of FcγRs was unaltered, regardless of cell separation procedure. Receptor expression was analysed in isolated leucocytes from anticoagulated peripheral blood by dextran sedimentation. Blood was mixed 1:1 with prewarmed 2% dextran (Polysciences Inc., Warrington, PA, USA) in RPMI medium (Sigma, Poole, UK) and incubated at 37°C for 30 minutes to sediment the erythrocytes preferentially. The white-cell supernatant was removed and centrifuged at 1,400 r.p.m. for 5 minutes; the cell pellet was washed in 1% BSA in PBS (prechilled to 4°C). The cell pellet was resuspended with staining buffer (prechilled to 4°C) to a concentration of 5 × 10^6 ^cells/ml. All subsequent staining procedures were conducted on ice.

### *In vitro *stimulation of leucocytes with fMet-Leu-Phe or tumour necrosis factor

Blood was incubated 1:1 with RPMI complete medium, comprising RPMI 1640, 10% heat-inactivated fetal bovine serum (FBS), 0.1 ml of sodium pyruvate, 2.0 mM L-glutamine, 25 mM 1 M HEPES, 0.1 mM non-essential amino acids, 0.25 μg/ml amphotericin, 50 μg/ml gentamicin and 50 μM 2-mercaptoethanol (all from Sigma) with or without pre-optimised concentrations of fMet-Leu-Phe (fMLP) (final concentration 100 nM) or TNF-α (100 U/ml; 10 ng/ml) for 0 minutes, 30 minutes, 1 hour or 4 hours at 37°C. After incubation, white blood cells were separated by the dextran sedimentation method described above and resuspended to 5 × 10^7^cells/ml for extracellular staining of the cells.

### Monoclonal antibodies

Murine monoclonal antibodies with the following specificities were used for primary stage staining: anti-FcγRIII (3g8), anti-FcγRII (iv.3), anti-NA1-FcRIIIb (CLBgran11) and anti-NA2-FcRIIIb (GRM1); all were gifts from Dr Paul Guyre (Dartmouth College, Hanover, NH, USA). CD62L and CD11b were purchased from Serotec (Oxford, UK). Secondary goat anti-mouse IgG F(ab')_2 _conjugated with fluorescein isothiocyanate was from CALTAG Labs (Burlingame, CA, USA).

### Staining procedure

Leucocytes were pipetted into a 96-well polypropylene plate (Costar, Cambridge, MA, USA) at a concentration of 250,000 per well. The plate was centrifuged (1,400 r.p.m. for 5 minutes at 4°C) and the supernatant was aspirated. The cells and reagents were kept at 4 to 8°C for the remainder of the experiment. To the cell pellet, blocking IgG (12 mg/ml Cohn Fraction II/III; Sigma) and specific primary antibody at 60 μg/ml were added and incubated for 45 minutes on ice. The cells were washed three times in staining buffer consisting of 0.2% BSA and 1 μg/ml sodium azide in PBS. The goat F(ab')_2 _anti-mouse IgG fluorescein isothiocyanate conjugate was then added, at 1 μg per well, for 30 minutes on ice. The cells were washed with staining buffer and the remaining erythrocytes were lysed; the leucocytes were fixed with BD Fix and Lyse reagent (Becton Dickinson). The cells were then washed twice more in staining buffer, resuspended to 200 μl in 1% paraformaldehyde and maintained at 4°C in the dark until analysis.

### Analysis of changes in antigen expression

Analysis by flow cytometry was performed with either a FACSCalibur or a FacScan analyser (Becton Dickinson). Forward and side-scatter gating removed contaminants such as cell debris. Data were acquired and analysed with the CellQuest^® ^application on a Power Macintosh G3 computer. Analysis of leucocyte subpopulations was completed with forward and side-scatter analysis. Monocytes were found to have high CD14 expression, neutrophils had low CD14 expression and lymphocytes were negative for CD14 expression. Expression of the receptors was correlated with the clinical measurements of disease made in the clinic, such as CRP, ESR and cell numbers. For each sample a minimum of 2,000 monocytes in the mixed leucocyte population were collected. The individual cell populations were then gated according to cell type and the specific median fluorescence intensity for each receptor was evaluated. Calibration was completed with Rainbow Microspheres (Sphereotech, Libertyville, IL, USA) to maintain consistency between experiments and to remove background fluorescence. Final values were expressed as milliequivalents of soluble fluorescein [40].

### Quantification and viability of leucocytes

Türk's solution, comprising 0.01% crystal violet in 3% acetic acid (Sigma), was prepared in distilled water and used for the enumeration of leucocytes. Trypan blue solution was used to determine the percentage of viable cells. Cells were resuspended in PBS, diluted 1:10 in trypan blue (0.4%; Sigma) and examined with a haemocytometer within 5 minutes of the addition of the dye. In addition to trypan blue staining, the apoptotic state of cells was assessed by the determination of hypodiploid DNA, because DNA breakdown is a hallmark of apoptosis. This was completed with propidium iodide as described previously [41]. In brief, cells were resuspended in 300 μl of PBS in 2% FBS (BioWhittaker, Walkersville, MD, USA) and permeabilised with 750 μl of ice-cold ethanol for 10 minutes at 4°C (BDH Laboratory Supplies, Poole, UK). Cells were then resuspended in 300 μl of PBS containing 50 μg/ml propidium iodide and 0.5 mg/ml RNAse A (Sigma). They were incubated for 20 minutes in the dark and washed once in PBS/FBS before analysis on a FACScan flow cytometer. Neutrophils were incubated overnight with 25 μg/ml etoposide (Sigma) as a positive control for apoptosis. There were no differences in the apoptotic state of isolated leukocytes.

### Generation of F(ab')_2_–biotin conjugates

Digestion of anti-FcγRII (iv.3) was completed with the ImmunoPure^® ^F(ab')_2_/Fab Ficin kit (Pierce, Rockford, IL, USA). This was found to have a greater percentage yield than pepsin digestion in preliminary studies, with no loss of avidity. Biotinylation of F(ab')_2 _was completed with a Sulfo-NHS-LC-Biotin kit (Pierce). Efficacy of biotinylation was assessed with the 4-hydroxyazobenzene-2-carboxylic acid (HABA) assay. To ensure that the affinity of the antibodies for the receptors was not compromised in any way after these series of procedures, an extracellular flow-cytometric staining assay was used to confirm binding. The biotinylation procedure was also assessed with streptavidin–phycoerythrin.

### Determination of reactive oxygen species

Oxidative burst was measured in neutrophils with the fluorochrome dihydrorhodamine (DHR)-123 (Sigma). This non-fluorescent and cell-permeable probe localises to the mitochondria, where it is converted into cationic DHR-123. It detects superoxide by reacting with hydrogen peroxide and/or peroxynitrite [42,43] to emit a 515 nm fluorescent signal when excited by a 488 nm argon-ion laser. DHR-123 was dissolved in dimethylsulphoxide to a concentration of 29 mM and stored in aliquots at -70°C. Neutrophils were isolated from peripheral blood by Ficoll–Histopaque density gradient centrifugation. Heparinised blood was mixed 1:1 with prewarmed RPMI 1640 and layered on a previously prepared step separation medium of equal volumes of Histopaque 1077 and Histopaque 1119 (Sigma). After centrifugation at 400 *g *for 30 minutes, the neutrophil layer at the 1119 and 1077 interface was carefully removed. Neutrophils were further purified by hypertonic lysis. The cell pellet was resuspended in ice-cold sterile water for 20 seconds followed by an equal volume of double-strength PBS to restore tonicity. They were resuspended to a final concentration of 5 × 10^6 ^cells/ml in 1% BSA/PBS and kept on ice until stimulation. Aliquots (50 μl) were combined with 200 μl of buffer consisting of saline (0.15 M) with 5 mM HEPES. DHR-123 was added to give a final concentration of 1 μM and the cells were incubated at 37°C for 5 minutes. After incubation, cells were stimulated with heat-aggregated IgG (HAIgG) (100 μg/ml) or by heterotypic or homotypic cross-linking of FcγRIIa and/or FcγRIIIb. Cross-linking was achieved by initial incubation of iv.3-B or 3g8-B (5 μg/ml) at 37°C for 5 minutes, as described by Vossebeld and colleagues [44] followed by various timed incubations with (Goat anti-mouse IgG (GAM); 50 μg/ml) or streptavidin (10 μg/ml). HAIgG was prepared by heating 1 mg/ml IgG in PBS at 63°C for 30 minutes, followed by centrifugation and aspiration of the supernatant. Preliminary experiments provided optimum concentrations of iv.3-B, 3g8-B, GAM, streptavidin and HAIgG to cause activation. In addition, the time of incubation was also determined. After incubation, reactions were stopped with a final concentration of 0.2% sodium azide; the cells were then placed on ice and analysed immediately by flow cytometry.

### Statistical analyses

Results are expressed as the arithmetic mean ± SEM for a given number of values *n*. Significant differences are defined is represented by a probability value, *p *< 0.05. Data was analysed with a Kolmogorov–Smirnov test for deviations from Gaussian distributions. All values were less than 0.2, *p *> 0.1; all data were therefore assumed to be normally distributed. Correlation was determined with linear regression to determine the line of best fit and with a two-tailed Pearson product moment correlation test to quantify how well *x *and *y *varied together. Statistical differences between two populations were analysed with either an unpaired *t*-test for data analysed for Gaussian distribution, or a two-tailed Mann–Whitney test for unpaired observations in all other cases. Kinetic distributions were analysed with either one-way or two-way analysis of variance with repeated measures. Significant changes (*p *< 0.05) were then analysed with the Bonferroni Dunn *post hoc *test that analyses associations between every combination of two parameters within the data. Analyses were completed with GraphPad Prism (version 3.00 for Windows; GraphPad Software, San Diego CA, USA) and Statview (version 5.0.1 for Windows; SAS Institute Inc., Cary, NC, USA). Gene frequency was determined with the Hardy–Weinburg formula.

## Results

### Surface expression of Fcγ receptors on peripheral leucocytes

#### Expression of FcγRIII

**Figure 1:**
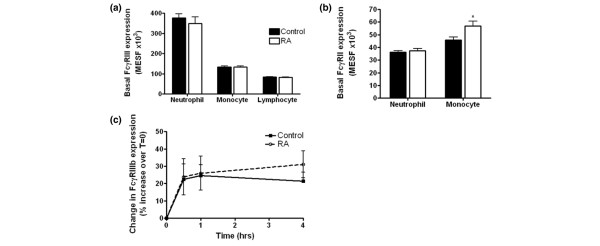
Expression of Fcγ receptors by rheumatoid and age/sex-matched control leucocytes. Baseline expression of FcγRIIIb **(a) **or FcγRIIa **(b) **in patients with rheumatoid arthritis (RA) and in controls. Blood was analysed for the baseline expression of FcγRIII on circulating leucocytes in an RA population and compared with a control. FcγRIIa was found to be higher on resting monocytes from patients with RA (*p *< 0.05; RA, *n *= 18; control, *n *= 18). Each experiment was performed in duplicate. **(c) **Change in FcγRIIIb expression on neutrophils in response to TNF-α. Two-way analysis of variance revealed no difference in the modulation of FcγRIIIb between RA and controls after stimulation with TNF-α. Results are expressed as the percentage change from the baseline expression (mean ± SE). Each experiment was performed in duplicate. MESF, milliequivalents of soluble fluorescein.

Analysis of receptor expression of basal peripheral leucocytes determined no significant difference between neutrophil FcγRIIIb expression in RA and control subjects (*p *> 0.05; Figure 1a). About 30% of monocytes expressed detectable levels of FcγRIIIa, detectable by the 3g8 antibody. There was no significant difference between either the level of expression (Figure 1a) or the percentage of cells expressing FcγRIIIa in the RA population in comparison with the control (data not shown); 10% of lymphocytes expressed FcγRIII. These were either natural killer cells or a T-lymphocyte subset. There was no difference in expression of FcγRIII in the overall lymphocyte population (Figure 1a). The gene frequency of NA1 and NA2 distribution in the sample population was comparable to that of European countries, white and black Americans and Tunisians (Table 2) [45-49]. The distributions of FcγRIIIb allotypes were similar in RA cases and controls.

**Table 2:** FcγRIIIb allotype gene frequency

Population	Gene frequency	
	
	NA1	NA2
Total	0.36	0.64
Control	0.39	0.61
RA	0.34	0.66

The Hardy–Weinburg formula was used to determine Fcγ receptor (FcγR)IIIb NA1 NA2 allotype gene frequency. There was no difference in distribution between RA and control populations (*p *> 0.05).

#### Expression of FcγRII

Low levels of the inhibitory receptor FcγRIIb have been determined in neutrophils [50]. It is not known whether this molecule is expressed on the surface and therefore contributes to FcγR-mediated signalling. Intact iv.3 antibodies detect trace amounts of FcγRIIb, but it has never been detected by using Fab fragments of iv.3, either by flow cytometry or in immunoprecipitation experiments (Susheela Tridandapani, personal communication). By using biotinylated iv.3 F(ab')_2 _fragments, specific for FcγRIIa, the potential impact of FcγRIIb was eliminated.

The baseline neutrophil expression of FcγRIIa did not differ between the two conditions and there was no correlation between resting receptor expression and any clinical measurement of disease (Figure 1b). However, examination of monocytic FcγRIIa revealed a higher resting expression RA than in controls (Figure 1b). Interestingly, there were no associations with any of the clinical disease activity parameters measured, which implies that the loss of FcγRIIa is not associated with severity of disease.

#### Fcγ receptor expression after stimulation

Peripheral whole blood from patients with RA or from control volunteers was incubated with TNF-α, fMLP or RPMI complete medium alone, as described in the Materials and methods section. Surface expression of FcγRIIIb increased after stimulation with TNF-α (Figure 1c), with no observable differences between RA and control samples. These results were identical to those observed after stimulation with fMLP (data not shown). In addition, after stimulation with either fMLP or TNF-α a 20 to 30% increase in surface expression of monocytic FcγRIIIa occurred, but this was not significantly different from the value at zero time (data not shown). There was no detectable modulation in surface expression of FcγRIIa on peripheral blood neutrophils or monocytes after stimulation with TNF-α or fMLP (data not shown).

### Measurements of neutrophil and systemic activation

#### Expression and regulation of L-selectin

**Figure 2:**
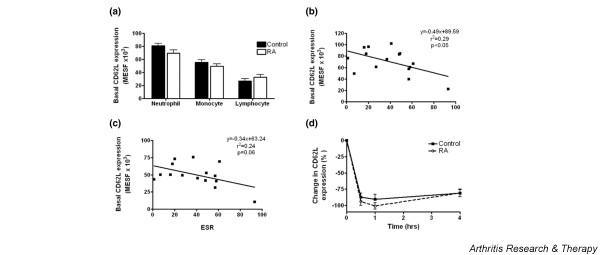
Expression of L-selectin by rheumatoid and age/sex-matched control leucocytes. **(a) **Baseline expression of L-selectin on peripheral leucocytes in patients with rheumatoid arthritis (RA) and control volunteers. Correlation of neutrophil **(b) **and monocyte **(c) **L-selectin and erythrocyte sedimentation rate (ESR). Linear regression of the relationship between resting neutrophil L-selectin expression and the ESR showed a significant correlation. Equation statistics are presented in the graph. In addition, Pearson's correlation also confirmed this finding (*r *= -0.54, *p *< 0.05; *n *= 15). Trends were also shown between monocyte L-selectin expression and ESR (0.1 > *p *> 0.05). **(d) **Neutrophil L-selectin was shed rapidly on TNF-α stimulation. MESF, milliequivalents of soluble fluorescein.

Examination of the basal expression of L-selectin on neutrophils showed a trend towards lower expression in patients with RA; however, this marginally failed to reach statistical significance (*p *= 0.07; Figure 2a). Examination of an association between basal L-selectin expression and ESR in patients with RA revealed a negative correlation between these variables (*R*^2 ^= 0.29, *p *< 0.05; Figure 2b). This suggests that the expression of L-selectin on neutrophils and monocytes decreases with the severity of the disease [36]. Correlations with other clinical markers of disease were not evident (data not shown). Analysis of L-selectin expression on monocytes revealed no difference between RA and control populations (Figure 2a). However, correlative analysis of ESR and basal L-selectin on monocytes revealed a similar trend of negative correlation to that for neutrophils (*R*^2 ^= 0.29, *p *= 0.06; Figure 2c). Between 50 and 80% of naïve and between 50 and 90% of memory CD3-positive T lymphocyte cells reportedly express L-selectin [51]. Basal expression of L-selectin did not differ between the two disease conditions (Figure 2a). Furthermore, comparisons with clinical blood data revealed no association of L-selectin expression on lymphocytes with any of the parameters measured, including ESR (data not shown).

After stimulation with TNF-α, a rapid decrease in expression of L-selectin within the first 30 minutes was detected (Figure 2d). Although receptor cleavage seemed to occur to a greater extent in RA neutrophils than in controls, this was not significant. The response to stimulation with fMLP was essentially identical to that with TNF-α incubation (data not shown). There were no statistical differences between stimulators or treatment groups at any time point (*p *> 0.05). On stimulation with TNF-α or fMLP, monocytic L-selectin expression was also shed. There was no observable difference between the RA and the control response for either treatment. Lymphocytes did not alter their expression of L-selectin in response to TNF-α or fMLP in either treatment group (*p *> 0.05; data not shown)

#### Expression and regulation of CR3

**Figure 3:**
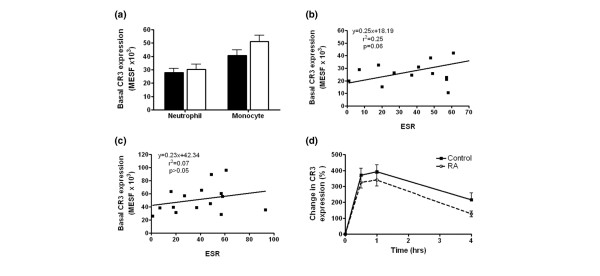
Expression of complement receptor type 3 (CR3) by rheumatoid and age/sex-matched control leucocytes. **(a) **Baseline expression of CR3 expression on peripheral leucocytes in Patients with rheumatoid arthritis and control volunteers. Correlation of neutrophil **(b) **and monocyte **(c) **CR3 and erythrocyte sedimentation rate (ESR). There was a trend for a positive correlation between neutrophil expression and ESR; however, this marginally failed to reach statistical significance (*p *= 0.06). **(d) **Neutrophil CR3 expression was upregulated in response to TNF-α. MESF, milliequivalents of soluble fluorescein.

Basal CR3 expression on neutrophils in patients with RA were no different from those in controls (Figure 3a). However, analysis of associations between clinical parameters of disease activity and basal CR3 expression revealed a trend towards a positive correlation between CR3 expression and ESR (Figure 3b). Analysis of CR3 expression on monocytes did not identify differences in baseline expression; neither was there any association with other indices of disease activity (Figure 3a, c, and data not shown).

In concordance with existing reports, the surface expression of CR3 on neutrophils increased after stimulation with either fMLP or TNF-α, but no difference was evident between control and RA populations (Figure 3c and data not shown). Examination of the TNF-α or fMLP-induced CR3 receptor upregulation on monocytes revealed no significant differences between the RA and control populations (data not shown).

### Analysis of reactive oxygen species

**Figure 4:**
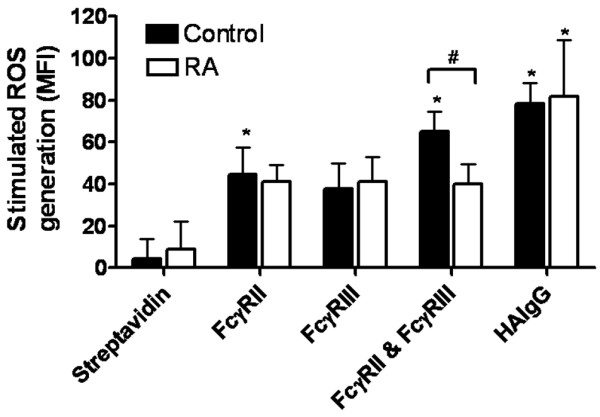
Stimulation of reactive oxygen species (ROS) in neutrophils by IgG Fcγ receptor engagement. Purified neutrophils were incubated with DHR-123 and stimulated with heat-aggregated IgG (HAIgG) or antibodies against IgG Fcγ receptors (FcγR) followed by streptavidin. Homologous cross-linking was completed by using iv.3–biotin (FcγRII) or 3g8–biotin (FcγRIII), and heterologous cross-linking by using both FcγRII and FcγRIII with streptavidin. Data are expressed at maximum fluorescence (45 minutes after stimulation). Experiments were performed in duplicate (*n *= 7). Comparisons between stimulations were completed by using a one-way analysis of variance with *post hoc *Bonferroni Dunn test. Significant differences from the streptavidin control are shown (*p < 0.05). Comparisons between rheumatoid arthritis and controls were performed by unpaired *t*-test (^#^*p *< 0.05). MFI, median fluorescence intensity.

Analysis of the intracellular release of ROS was determined with DHR-123. Purified neutrophils were incubated with DHR-123 before specific stimulation, and the median fluorescence intensity was determined. Analysis was completed at the maximum detectable fluorescence intensity, which was 45 minutes after stimulation. Figure 4 demonstrates the maximal ROS generation by neutrophils from IgG Fc receptor engagement with the use of different stimuli, in control and RA subjects. Specific ligation of either FcγRIIa or FcγRIIIb produced an observable increase in ROS, although this reached statistical significance only for FcγRIIa ligation in the control group, where ROS production was higher after FcγRIIa ligation, in comparison with FcγRIIIb. Heterologous cross-linking of FcγRIIa and FcγRIIIb induces ROS production to a greater extent than engagement of either receptor alone (Figure 4). This is consistent with the current data demonstrating that heterotypic FcγRIIIb–FcγRIIa co-ligation produces enhanced neutrophil activation in terms of phagocytosis, oxidative burst and release of granular enzymes [44,52]. In addition, HAIgG has maximal efficiency in producing ROS. This is probably due to spatial orientation of the HAIgG molecule, which favours clustering of a greater number of receptors on the cell. In these circumstances, the differential ROS generation between control and RA groups was not apparent.

Comparisons between the control and RA samples revealed that the resting generation of ROS by untreated cells was marginally higher in the RA population than in the controls, but this was not significant (data not shown). There was no difference in ROS generation as a result of single FcγR ligation between the two treatment groups. However, dual ligation of FcγRIIa and FcγRIIIb resulted in an additive effect in control populations that was absent from the RA group (*p *< 0.05, unpaired *t*-test). This loss of ROS generation within the RA group was not correlated with age or with disease severity as measured by physician-assessed DAS 28 (data not shown). Furthermore, the sample size was insufficient to determine effects of gender on loss of ROS generation.

## Discussion

We have demonstrated that there is a defect in the cooperative effect of FcγRIIa and FcγRIIIb in mediating ROS generation in neutrophils. This was not due to a change in receptor expression, because basal levels of both receptors were equivalent in control and RA populations, which is consistent with previous reports [51,52]. It is also unlikely that it results from an aberrant response in the direct downstream signalling pathways for each individual receptor. Because the efficacy of HAIgG-induced ROS was not altered, the disparities must lie in the conformational changes of the receptors during cross-linking, which results from antigen binding. The generation of ROS is pivotal in the efficient destruction of foreign material [53,54]. The reduction in ROS generation may therefore account in part for the increased susceptibility and morbidity associated with infection in RA [27,29,30,53]. However, the extent of this FcγR-dependent defect is limited because individuals with FcγRIIIb deficiency do not suffer from recurrent bacterial infections and because removal of the FcγRIIIb does not eliminate the phagocytic capacity and subsequent destruction of opsonised bacteria [20,55,56]. We suggest that, in cases where the pathogenic agonist is less efficient in the induction of cross-linking, this becomes an impeding factor in the generation of ROS.

Published data on neutrophil Fc-mediated ROS generation are conflicting. We demonstrated that the efficacy of ROS generation by homotypic ligation did not differ between FcγRIIa and FcγRIIIb, which is consistent with two other studies [17,38]. This is contrary to the work of Hundt and Schmidt [37] showing that FcγRIIIb induces a greater oxidative burst than FcγRIIa. This may be due to the high concentration of GAM used in their experiments and subsequent non-specific ligation, particularly because studies with smaller amounts of GAM do not show this difference in efficacy [38]. In our studies we generated biotinylated F(ab')_2 _anti-FcγRIIa and FcγRIIIb molecules and used streptavidin for cross-linking; the potential for non-specific ligation of receptors, which can occur when using a secondary IgG, was therefore eliminated. Initial optimisation studies in the work presented here did not demonstrate any increase in fluorescence with a concentration twofold higher for primary or secondary reagents (data not shown). Furthermore, the concentrations were comparable to those used in other assays, which activate neutrophils by specific ligation [15,17,44]. Aside from the controversy about the relative efficacy of FcγRII and FcγRIIIb in ROS generation, early studies could not produce an FcγRIIIb-mediated oxidative burst [57,58]. These investigations used a different monoclonal antibody (CBL-FcR-gran-I) against FcγRIIIb in comparison with all other studies mentioned here (3g8). Antibodies 3g8 and Gran-1 recognise different epitopes within the ligand-binding site of FcγRIIIb [59]. This may result in differences in receptor aggregation, thereby affecting subsequent signalling transduction pathways.

Our analysis of cellular markers of activation, L-selectin and CR3, also suggest that the neutrophils of patients with RA in the periphery are activated before joint infiltration. Furthermore, the negative correlation between neutrophil L-selectin expression and ESR suggests that neutrophil activation increases with disease severity. The altered expression in adhesion molecules may account for the defective migratory capacity of neutrophils to inflammatory targets observed in patients with RA [60]. Although several studies have shown that L-selectin is lower on neutrophils from synovial fluid, there are fewer observations for neutrophils from peripheral blood. Three studies report no change; however, Bond and colleagues have shown a decreased level of L-selectin on circulating granulocytes [61-64]. Previous studies have also failed to determine a difference in neutrophil CR3 expression in peripheral blood in RA in comparison with controls; however, upregulation is widely reported in synovial neutrophils [61,63,65]. The disparity in the data probably arises from inter-patient variability, small sample size, and requirements for study recruitment, together with the use of cell separation techniques that downregulate L-selectin and upregulate CR3. In the studies presented here, neutrophils were stimulated with an optimum concentration of fMLP or TNF-α to examine any differences in the shedding response of L-selectin. There was no difference in L-selectin shedding between RA and controls. However, further preliminary work has shown that the loss in receptor expression by TNF-α is dose-dependent and that neutrophils from patients with RA require a lower concentration of TNF-α for equivalent shedding than do those in control samples. An earlier study has shown that TNF receptor expression is equivalent in patients and controls, reducing the probability that this is a cause of the effect [66].

The studies presented here demonstrate that functional abnormalities exist in peripheral neutrophils from patients with RA. This defect resides in the capacity of neutrophils to generate ROS in response to cooperative ligation of FcγRIIa and FcγRIIIb. The decreased production of ROS is unrelated to the level of receptor expression. This, together with the altered expression of adhesion molecules, may account for the increase in susceptibility and morbidity to bacterial infections that exists in RA.

## Conclusion

This study demonstrates that patients with active RA have an altered capacity of generating ROS in response to dual ligation of FcγRII and FcγRIIIb. This may be a compensatory mechanism to downregulate the response to self ICs, and may affect the response to bacterial infections.

## Abbreviations

BSA = bovine serum albumin; CR3 = complement receptor type 3; CRP = C-reactive protein; DHR = dihydrorhodamine; ESR = erythrocyte sedimentation rate; FBS = fetal bovine serum; FcγR = Fcγ receptor; fMLP = fMet-Leu-Phe; GAM = goat anti-mouse IgG; HAIgG = heat-aggregated IgG; PBS = phosphate-buffered saline; ROS = reactive oxygen species; TNF = tumour necrosis factor.

## Competing interests

The authors declare that they have no competing interests.

## Authors' contributions

AMF co-designed the study, completed the experimental work and wrote the paper. PKW aided development of the reagents and co-designed the study. ASMJ contributed samples and discussion of the study. NJG co-designed the study, gave overall supervision and provided funding and editing of the paper. All authors read and approved the final manuscript.
